# Atherosclerotic Aortic Calcification-Associated Polymorphism in *HDAC9* and Associations with Mortality, Cardiovascular Disease, and Kidney Disease

**DOI:** 10.1016/j.isci.2020.101253

**Published:** 2020-06-10

**Authors:** Johan Ärnlöv, Douglas F. Dluzen, Christoph Nowak

**Affiliations:** 1 Department of Neurobiology, Care Sciences and Society (NVS), Family Medicine and Primary Care Unit, Karolinska Institutet, Alfred Nobels Allé 23, Huddinge 14183, Sweden; 2 School of Health and Social Studies, Dalarna University, Falun 79188, Sweden; 3 Department of Biology, Morgan State University, Baltimore, MD 21251, USA

**Keywords:** Medical Specialty, Clinical Finding, Genomics

## Abstract

Histone deacetylase 9 (HDAC9) has recently been demonstrated as a key regulator of vascular smooth muscle cell (VSMC) phenotype and is associated with abdominal aortic calcification, myocardial infarction, and ischemic stroke. It is uncertain whether HDAC9 is also implicated in other VSMC-driven diseases. Our objective was to assess associations between abdominal aortic calcification-associated genetic variation in *HDAC9* and VSMC-associated phenotypes. In this prospective population study of 335,146 adults enrolled in the UK Biobank, the abdominal aortic calcification-associated risk allele of a genetic variant in *HDAC9* was associated with increased risk of systolic hypertension, non-ST segment elevation myocardial infarction, and ischemic stroke. There was a suggestive protective association with kidney disease outcomes that did not reach experiment-wise significance. These genetic results lend further support for HDAC9 as a potential therapeutic target for arterial stenotic and calcific disease.

## Highlights

• AAC-associated variation in *HDAC9* was associated with hypertension, STEMI, and stroke
• A suggestive protective association with kidney function was not significant
• HDAC9 could be explored as a therapeutic target for arterial calcific disease

## Graphical Abstract

**Figure:**
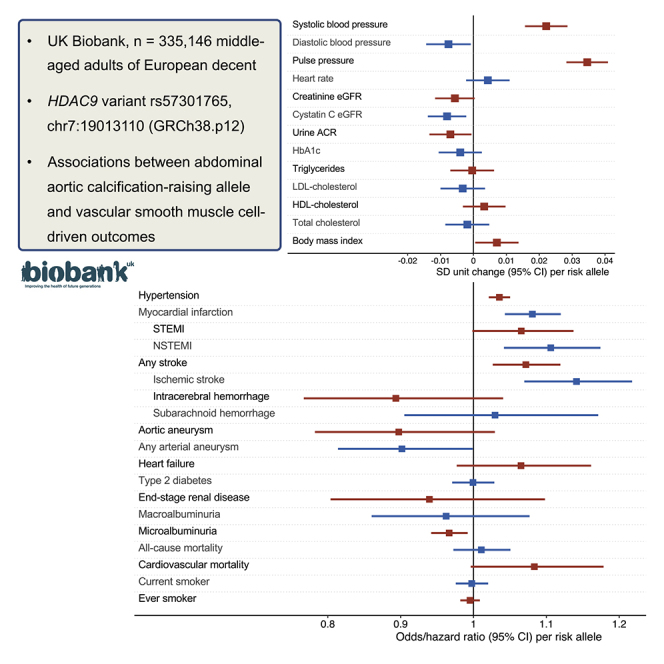

## Introduction

In a recent translational genomics study, Malhotra et al. (2019) identified the first genetic risk locus for abdominal aortic calcification (AAC) in *HDAC9* on chromosome 7, which encodes histone deacetylase 9 (HDAC9), a co-regulator of gene transcription. In genome-wide association study (GWAS) and *in vitro* studies, the authors demonstrated HDAC9 as a key regulator of vascular smooth muscle cell (VSMC) phenotype and calcification. Mice deficient in HDAC9 had reduced vascular calcification, suggesting HDAC9 as a possible drug target for vascular calcific disease (Malhotra et al., 2019). A separate study found that HDAC9 inhibition in mice reduced arterial neointimal formation and improved stenotic disease (Lino Cardenas et al., 2019). These findings suggest HDAC9 as a potential drug target for VSMC-associated cardiovascular disease.

Arterial calcification is a risk factor for cardiovascular disease and mortality (Bastos Goncalves et al., 2012, Criqui et al., 2014) and is associated with hypertension, chronic kidney disease, and end-stage renal disease (Vervloet and Cozzolino, 2017). The results obtained by Malhotra et al. (2019) demonstrate HDAC9's role in altering VSMC phenotype, and associations with myocardial infarction, ischemic stroke, and pulse pressure have been reported (Traylor et al., 2012, Bellenguez et al., 2012, Malik et al., 2016, Malik et al., 2017, Nikpay et al., 2015, Kato et al., 2015). However, it is uncertain whether HDAC9 is also implicated in other VSMC-driven diseases. Investigating these associations can further delineate the potential role of HDAC9 as a treatment target in arterial calcific and stenotic disease. Here, we analyzed a population sample of 335,146 adults to assess the role of *HDAC9* in mortality and VSMC-driven cardiovascular and renal pathology.

## Results

### Associations in the UK Biobank

**Figure 1:**
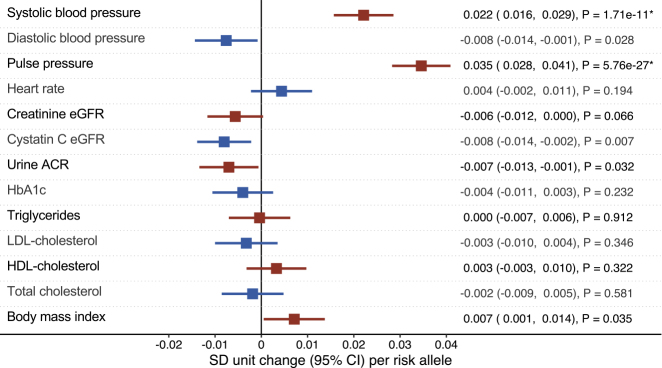
Forest Plot of Per-Risk-Allele Associations with Continuous Outcomes Associations represent the change in standard deviation unit per added AAC-raising risk allele. Horizontal lines denote 95% confidence intervals. Bonferroni significance is marked by asterisks.

**Figure 2:**
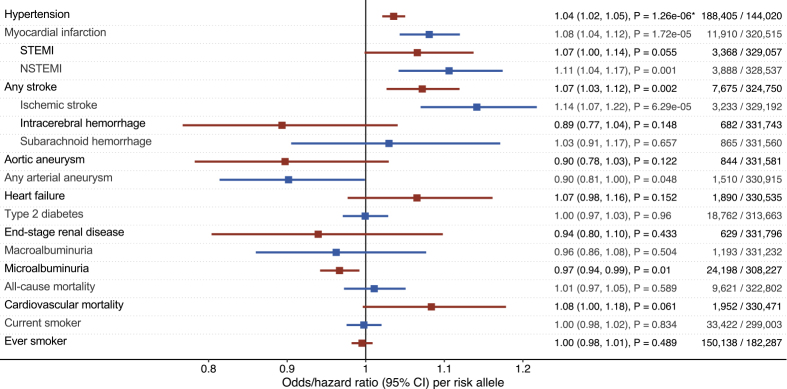
Forest Plot of Per-Risk-Allele Associations with Binary Outcomes Effects per added AAC-raising risk allele are shown as odds ratios or hazard ratios (for all-cause mortality and cardiovascular mortality). Horizontal lines denote 95% confidence intervals. Bonferroni significance is marked by asterisks.

Among 335,146 participants, the AAC-associated single nucleotide polymorphism rs57301765 was genotyped in 332,425 persons (99.19%, 53.7% women, age 56.9 ± 8.0 years, 10.1% smokers). We ascertained genotyping quality as detailed in the Supplemental Information (Figures S1–S4, Tables S1 and S2). Maximum follow-up was 10.0 years (mean 7.0 ± 1.0, median 7.1, interquartile range, 6.4–7.8 years). Figures 1 and 2 display associations between the AAC-associated allele and outcomes. At experiment-wise significance, we found associations between rs57301765 and systolic blood pressure (p = 1.71 × 10^−11^), pulse pressure (p = 5.76 × 10^−27^), and hypertension (p = 1.26 × 10^−6^). The associations with myocardial infarction (p = 1.72 × 10^−5^; driven by non-ST segment elevation myocardial infarction [NSTEMI], p = 0.001) and ischemic stroke (p = 6.29 × 10^−5^) were not significant at the strict Bonferroni level (Figures 1 and 2). There was no clear association between *HDAC9* and aneurysmal disease, but there was a nominally significant protective association with diastolic blood pressure (p = 0.028). We found suggestive directionally consistent protective associations between the AAC-raising variant and kidney disease outcomes that reached nominal (but not experiment-wise) significance: microalbuminuria (p = 0.010), albumin-to-creatinine ratio (ACR, p = 0.032), and estimated glomerular filtration rate based on cystatin C levels (cystatin C-eGFR, p = 0.007).

### Associations in Previously Reported GWAS Meta-Analyses

Results from previously reported GWAS meta-analyses (Table S2) confirm our findings in the UK Biobank sample in that the AAC risk allele was associated with raised pulse pressure (p = 4.00 × 10^−6^) and hypertension (p = 0.005). There was no association with diastolic blood pressure (p = 0.728) or aortic valve calcification (p = 0.767). We found a nominally significant protective effect on ACR risk (p = 0.037, Table S2). The non-significant (p = 0.152) risk-increasing association with heart failure risk in our sample corresponded to a nominal effect in the same direction (p = 0.015) in the HERMES heart failure consortium.

## Discussion

In a population sample of >330,000 adults, we used a genetic variant to query the potential effects of targeting HDAC9 for the treatment of VSMC-associated diseases. We discovered associations between the AAC-raising variant and increased systolic hypertension and raised risk of NSTEMI that confirm previously reported effects on cardiovascular outcomes. A suggestive protective association with kidney disease phenotypes did not reach experiment-wise significance but was directionally consistent across renal endpoints. We confirmed associations with ischemic stroke previously reported in independent samples. There were no clear associations with metabolic cardiovascular risk factors such as plasma lipid and glucose levels, or mortality.

The stronger association between *HDAC9* and NSTEMI, compared with ST segment elevation myocardial infarction (STEMI), could suggest *HDAC9* acting on myocardial infarction risk primarily through non-atherosclerotic mechanisms, as a higher proportion of NSTEMI can be attributed to non-atherosclerotic causes (Zipes et al., 2019). Whether arterial calcification is a particular risk factor for NSTEMI has not been assessed before. The causes of myocardial oxygen demand versus consumption mismatch in NSTEMI include stenotic disease and systemic pathologies such as hypertension (Zipes et al., 2019). Our results suggest a role of HDAC9 in myocardial infarction through non-coronary plaque-related, possibly stenotic, hypertensive, or calcific aortic pathology. However, the differential associations with STEMI and NSTEMI in our study need to be interpreted with great caution as (1) the clinical diagnoses in the UK Biobank have not been validated for accuracy, (2) our study may be underpowered (e.g., the effect size of 1.07 and p-value of 0.055 for STEMI suggests that larger samples may detect an effect that we missed because of lack of power), and (3) although non-atherosclerotic flow-limiting causes (such as systemic arterial hypertension) are more common for NSTEMI, both STEMI and NSTEMI share atherosclerosis as the predominant cause. Hence, our discovery of an apparently stronger effect of AAC-raising genetic variation in *HDAC9* on NSTEMI versus STEMI risk points to an interesting distinction that has received little attention in previous research. Yet, the limitations of our study do not allow firm conclusions, and additional studies exploring a potential differential role of HDAC9-driven AAC in different cardiovascular pathologies are needed.

The strong association with systolic hypertension confirms HDAC9 as a likely key regulator of VSMC-related cardiovascular diseases (Malhotra et al., 2019, Lino Cardenas et al., 2019). The suggestive protective effect on kidney disease due to AAC-associated genetic variation in our comparatively healthy sample has not been reported before and requires independent replication. Although we found consistent effect sizes in the protective direction across renal phenotypes, none of the associations reached experiment-wise significance. Our sample comprising comparatively healthy, middle-aged adults was likely underpowered to detect potential effects on renal outcomes (the proportion of participants with eGFR below 60 was 2.2%, and 58.2% had normal kidney function with an eGFR above 90).

We found no association between HDAC9 and several conventional cardiovascular risk factors (apart from blood pressure traits), which supports HDAC9 as a possible add-on drug target for cardiovascular disease acting through different mechanisms than established therapies. Whether pharmacological targeting of HDAC9 could enhance existing blood pressure treatments or act as an alternative treatment remains uncertain and needs to be addressed in future experimental studies.

### Limitations of the Study

Limitations include exclusive European ancestry, limited power for some outcomes (which may have missed true effects), reliance on a single genetic variant, and the caveats of extrapolating from genetic effects to clinical reality. The clinical phenotypes in the UK Biobank have not been validated for clinical accuracy (leaving doubts about the distinction between NSTEMI and STEMI based on hospital admissions records), and there is evidence for a “healthy selection bias” in the UK Biobank. Our epidemiologic study provides limited mechanistic insights, and cardiovascular risk groups such as elderly persons or those with chronic kidney disease were likely underrepresented. Strengths include the large, contemporary sample representative of the UK population, consistent case ascertainment, exclusion of genotyping errors, and confirmation in independent GWAS data.

### Conclusion

In a community sample of >330,000 adults, we found confirmatory evidence in support of HDAC9 as a key regulator and potential therapeutic target for VSMC-driven cardiovascular disease, with suggestive evidence of a reverse effect on kidney disease.

### Resource Availability

#### Lead Contact

Dr. Christoph Nowak, M.D., Ph.D., Dipl.-Psych., Department of Neurobiology, Care Sciences and Society (NVS), Karolinska Institutet, Alfred Nobels Allé 23, SE-14183 Huddinge, Sweden, email: christoph.nowak@ki.se, phone: +46–739806535, ORCID iD: 0000-0001-8435-3978.

#### Materials Availability

We used existing data as described below. No new data or materials were generated in our study.

#### Data and Code Availability

Researchers can apply to use the UK Biobank data for health-related research in the public interest (http://www.ukbiobank.ac.uk/register-apply/). After ethical approval, the UK Biobank releases de-identified data to approved researchers for specific research projects. All the data used obtained through look-ups of GWAS repositories are in the public domain and available via the webpages provided in the main text and Table S2. The analysis code can be obtained from the corresponding author (C.N.) on request.

## Methods

All methods can be found in the accompanying Transparent Methods supplemental file.

## Supplemental Information

Document S1. Transparent Methods, Figures S1–S4, and Tables S1 and S2

## Footnotes

Supplemental Information can be found online at https://doi.org/10.1016/j.isci.2020.101253.
