# Supplementary material for: Atherosclerotic Aortic Calcification-Associated Polymorphism in HDAC9 and Associations with Mortality, Cardiovascular Disease, and Kidney Disease
Source: iScience. 2020 Jun 10;23(7):101253. doi: 10.1016/j.isci.2020.101253 (PMC7322070; doi:10.1016/j.isci.2020.101253)
Supplement: Document S1. Transparent Methods, Figures S1–S4, and Tables S1 and S2 [file mmc1.pdf]

iScience, Volume *23*

Supplemental Information

Atherosclerotic Aortic Calcification-Associated Polymorphism in *HDAC9* and Associations with Mortality, Cardiovascular Disease, and Kidney Disease

Johan Ärnlöv, Douglas F. Dluzen, and Christoph Nowak

Transparent Methods

Study rationale

Large population studies are valuable for querying the predicted effects of therapeutic targeting of proteins. Genetic variants that mimic manipulation of the target protein act over the entire lifespan and can serve as proxies to assess expected long-term effects. As genetic variants are randomly allocated at conception, any non-random differences between carriers and non-carriers of an exposure-associated variant should be attributable to different genotypes. Carriers of a variant known to influence an outcome through a specific protein can therefore provide insights into the possible effects of manipulating the protein pharmacologically (Plenge et al., 2013, Nguyen et al., 2019). Important caveats include ignoring biological canalization, sensitive periods, selection pressure, threshold effects, and off-target effects. Although altering protein activity in adult life can have different consequences than genetically determined activity, querying epidemiologic studies has proven useful in predicting drug effects (Schmidt et al., 2019).

Study population

The UK Biobank (http://www.ukbiobank.ac.uk) recruited 2006-2010 altogether 502,655 persons aged 40-69 years from the UK population. Informed consent was followed by standardized health assessments and follow-up across national health registers. We included all participants with genotyping data included in genetic principle component analysis (excluding related individuals) and assigned to "white British" ethnicity. Participants who had withdrawn consent by August 2019 were excluded. Maximum follow-up was until 31 March 2017. We selected the following VSMC-associated outcomes (requiring >500 cases): blood and pulse pressure, heart rate, hypertension, myocardial infarction, stroke, aortic/arterial aneurysm, heart failure, estimated glomerular filtration rate (eGFR) by serum creatinine or cystatin C, urine albumin-to-creatinine ratio (ACR), end-stage renal disease, all-cause and cardiovascular mortality, glycated hemoglobin (HbA1c), diabetes, serum lipids, body mass index, and smoking history. Disease endpoints were defined based on nurse interview, hospital episode diagnoses (ICD-9/10), and medication records (**Table S1**).

Representativeness of the sample

UK Biobank participants are more likely to be female, older, healthier, and less exposed to socioeconomic deprivation than the general UK population (Fry et al., 2017). The limited number of persons with genotyping data who reported non-European ancestry required us to exclude these participants from the analysis. The Generalizability of our results to non-European ethnicities is therefore uncertain.

Statistical analysis

We selected the top single nucleotide polymorphism (SNP) rs57301765 (hg38 chr7:19,052,733) in HDAC9, whose minor A-allele was associated with worse AAC (Malhotra et al., 2019). Out of six reported AAC-associated SNPs in HDAC9 (Malhotra et al., 2019), only rs57301765 was directly genotyped in the UK Biobank.

We queried public GWAS data using PhenoScanner (http://www.phenoscanner.medschl.cam.ac.uk/). We also performed look-ups in the HERMES heart failure (http://www.hermesconsortium.org/mou) and CKDGen chronic kidney disease (https://ckdgen.imbi.uni-freiburg.de/) consortia to identify genetic associations in addition to the previously reported associations with ischemic stroke (METASTROKE) and myocardial infarction (CARDIoGRAMplusC4D) (Malik et al., 2016, Malik et al., 2017, Nikpay et al., 2015).

We used logistic and linear regression adjusted for age, sex, genotyping array, and the top ten genetic principle components to assess associations between the AAC-associated allele and outcomes. Adjusted Cox regression was used for mortality, and proportional hazards and linearity ascertained in Schoenfeld and Martingale residual plots. Skewed distributions of HbA1c and ACR prompted natural log-transformation to normality. We used Bonferroni (experiment-wise) correction for multiple comparisons adjusting for a total number of phenotypes in the UK Biobank of about 7,500 ($P < 5\times10^{-6}$). Analyses were carried out in R v.3.3.

Ethical approval

The UK Biobank study was approved by the North West Multi-Centre Research Ethics Committee. The current study (UK Biobank project 42176) complies with the Declaration of Helsinki and ethical approval was granted by the Swedish Ethical Review Authority (Dnr. 2019-02328). Data from all participants who withdrew consent were excluded from the study. The purpose of this study was approved by the UK Biobank organization in accordance with participant consent as representing health-related research in the public interest.

Patient and public involvement

The purpose of this study was approved by the UK Biobank organization in accordance with participant consent as representing health-related research in the public interest. We did not directly involve patients or the general public in this study.

Data sharing information

Researchers can apply to use the UK Biobank data for health-related research in the public interest (http://www.ukbiobank.ac.uk/register-apply/). After ethical approval, the UK Biobank releases de-identified data to approved researchers for specific research projects. All the data used obtained through look-ups of GWAS repositories are in the public domain and available through the webpage provided in the main text and **Table S2**. The analysis code can be obtained from the corresponding author (C.N.) on request.

Ascertainment of genotyping quality

Concerns about the genotyping quality of a specific genetic variant in the UK Biobank recently led to a very prominent example of a retracted publication in *Nature Medicine* (Wei and Nielsen, 2019). In a series of analyses published independently by three different research groups and published on the preprint server *biorxiv*, it was demonstrated that poor genotyping performance on the UK Biobank arrays and technical error plausibly led to erroneous results that could not be replicated in other samples (Gudbjartsson et al., 2019, Maier et al., 2019, Tanigawa and Rivas, 2019). In order to ascertain the genotyping quality of the variant rs57301765 (hg38 chr7:19,052,733) in the UK Biobank, we followed the procedure by Yosuke Tanigawa and Manuel Rivas (2019). The variant rs57301765 was genotyped on both the UK Biobank Affymetrix array (n = 95 batches) and the UKBiLIEVE array (n = 11 batches). None of the batches failed genotyping for this variant. The overall genotyping missingness rate of 0.81% in our sample of 335,146 White British individuals was acceptably low. There were no systematic differences between the arrays and no outlying genotyping batches were apparent with regard to genotyping rate, which ranged from 98.08% - 99.69% (median 99.25%), and 98.20% - 99.30% (median 98.68%) for the Affymetrix and UKBiLIEVE arrays, respectively. Cluster plots and additional genotyping performance data were obtained using the *ScatterShot* application by Mark McCarthy's group (http://mccarthy.well.ox.ac.uk/ScatterShotWebApp/ui/scattershot). We selected the sample of 409,634 White British individuals in *ScatterShot*. **Figure S1** shows cluster plots of genotype alternatives for women and men, demonstrating acceptable separation of genotype classes.

As the *P*-value for Hardy-Weinberg equilibrium (HWE) implied deviations from HWE ($P_{women} = 2.9\times10^{-8}$, $P_{men} = 8.2\times10^{-8}$), we assessed whether poor genotyping quality across batches or arrays could explain the deviation from HWE. As in the publication from Manual Rivas' group (Tanigawa and Rivas, 2019), we extracted genotyping rate, batch and array identifiers, heterozygosity rate, and HWE *P*-values for each batch. As illustrated in **Figure S2**, we found no association between $-\log_{10}$(HWE *P*-value) and either array (linear regression $P = 0.141$), or batch ($P = 0.491$).

The distributions of $-\log_{10}$(HWE *P*-value) did not differ between arrays (Kolmogorov-Smirnov test, $P = 0.412$), or between batches with genotyping rates above or below the median ($P = 0.911$) (**Figure S3, Figure S4**). Finally, the minor allele frequency in our sample of 0.1532 was comparable to the minor allele frequency of 0.1696 in the European non-Finnish reference sample reported on the gnomAD browser (http://gnomad.broadinstitute.org/).

Taken together, these data demonstrate good genotyping performance for rs57301765 in the UK Biobank. Technical error or inadequate quality control were not apparent and are unlikely to explain the possible deviation from Hardy-Weinberg equilibrium.

Supplemental References

Plenge, R.M., Scolnick, E.M., and Altshuler, D. (2013). Validating therapeutic targets through human genetics. Nat. Rev. Drug. Discov. *12*, 581-594.

Nguyen, P.A., Born, D.A., Deaton, A.M., Nioi, P., and Ward, L.D (2019). Phenotypes associated with genes encoding drug targets are predictive of clinical trial side effects. Nat. Commun. *10*, 1579.

Schmidt, A.F., Holmes, M.V., Preiss, D., et al (2019). Phenome-wide association analysis of LDL-cholesterol lowering genetic variants in PCSK9. BMC Cardiovasc. Disord. *19*, 240.

Fry, A., Littlejohns, T.J., Sudlow, C., Doherty, N., Adamska, L., Sprosen, T., Collins, R., and Allen, N.E. (2017). Comparison of sociodemographic and health-related characteristics of UK Biobank participants with those of the general population. Am. J. Epidemiol. *186,* 1026-1034.

Malhotra, R., Mauer, A.C., Lino Cardenas, C.L., et al (2019). HDAC9 is implicated in atherosclerotic aortic calcification and affects vascular smooth muscle cell phenotype. Nat. Genet. *51*, 1580-1587.

Malik, R., Traylor, M., Pulit, S.L., et al (2016). Low-frequency and common genetic variation in ischemic stroke: The METASTROKE collaboration. Neurology *86*, 1217-1226.

Malik, R., Dau, T., Gonik, M., et al (2017). Common coding variant in SERPINA1 increases the risk for large artery stroke. *Proc. Natl. Acad. Sci. U S A. 114*, 3613-3618.

Nikpay, M., Goel, A., Won, H.H., et al (2015). A comprehensive 1,000 Genomes-based genome-wide association meta-analysis of coronary artery disease. *Nat. Genet. 47*, 1121-1130.

Wei, X., and Nielsen, R. (2019). Retraction Note: CCR5-Δ32 is deleterious in the homozygous state in humans. Nat. Med. *25*, 1796.

Gudbjartsson, D., Sulem, P., Stefansson, K., Mars, N., Karjalainen, J., Ripatti, S., Palotie, A., and Daly, M. (2019). CCR5-del32 is not deleterious in the homozygous state in humans. bioRxiv. 2019:788117. https://doi.org/10.1101/788117. Accessed December 1, 2019.

Maier, R., Akbari, A., Wei, X., Patterson, N., Nielsen, R., and Reich, D. (2019). No statistical evidence for an effect of CCR5-Δ32 on lifespan in the UK Biobank cohort. bioRxiv. 2019:787986. https://doi.org/10.1101/787986. Accessed December 1, 2019.

Tanigawa, Y., and Rivas, M.A. (2019). Reported CCR5-Δ32 deviation from Hardy-Weinberg equilibrium is explained by poor genotyping of rs62625034. bioRxiv. 2019:791517. https://doi.org/10.1101/791517. Accessed December 1, 2019.

Table S1. Outcome definitions in the UK Biobank sample, Related to Figure 1 and Figure 2.

Variable	UK Biobank data field and/or study-specific definition	Additional comments
**Systolic blood pressure**	http://biobank.ctsu.ox.ac.uk/showcase/field.cgi?id=4080	Automated reading, two measures taken a few moments apart. OMRON Healthcare BP device.
**Diastolic blood pressure**	http://biobank.ctsu.ox.ac.uk/showcase/field.cgi?id=4079	Automated reading, two measures taken a few moments apart. OMRON Healthcare BP device.
**Hypertension**	Self-reported diagnosis of hypertension, current blood pressure medication, physician diagnosis of hypertension, SBP >140 mmHg or DBP >90mmHg	
**Heart rate**	http://biobank.ctsu.ox.ac.uk/showcase/field.cgi?id=95	(during blood pressure assessment)
**Pulse pressure**	SBP minus DBP	
**Stroke**	http://biobank.ctsu.ox.ac.uk/showcase/field.cgi?id=42006	Centrally algorithmically defined by UK Biobank
**Ischemic stroke**	http://biobank.ctsu.ox.ac.uk/showcase/field.cgi?id=42008	Centrally algorithmically defined by UK Biobank
**Intracerebral hemorrhage**	http://biobank.ctsu.ox.ac.uk/showcase/field.cgi?id=42010	Centrally algorithmically defined by UK Biobank
**Subarachnoid hemorrhage**	http://biobank.ctsu.ox.ac.uk/showcase/field.cgi?id=42012	Centrally algorithmically defined by UK Biobank
**Myocardial infarction**	http://biobank.ctsu.ox.ac.uk/showcase/field.cgi?id=42000	Centrally algorithmically defined by UK Biobank
**STEMI**	http://biobank.ctsu.ox.ac.uk/showcase/field.cgi?id=42002	Centrally algorithmically defined by UK Biobank
**NSTEMI**	http://biobank.ctsu.ox.ac.uk/showcase/field.cgi?id=42004	Centrally algorithmically defined by UK Biobank
**Aortic aneurysm**	ICD-9: 441, ICD-10: I71	
**Any arterial aneurysm**	ICD-9: 441, 442, 414.1, 417.1, 437.3; ICD-10: I71, I72, I79.0, I25.3, I25.4, I28.1, I67.1	
**Creatinine eGFR**	Serum creatinine measured by enzymatic assay (Beckman Coulter, UK) on a Beckman Coulter AU5800 analyzer.	CKD-EPI equation adjusted for sex, ethnicity and age
**Cystatin C eGFR**	Serum cystatin C measured by immuno-turbidimetric assay (Siemens, Germany) on a Siemens ADVIA 1800 analyzer	CKD-EPI equation adjusted for sex and age
**Urine albumin to creatinine ratio (ACR)**	http://biobank.ctsu.ox.ac.uk/showcase/field.cgi?id=30510 http://biobank.ctsu.ox.ac.uk/showcase/field.cgi?id=30500	Urine microalbumin measured by immuno-turbidimetric assay (Randox Bioscience, UK) on an AU5400 analyzer. Values below analytical range 6.7-200 mg/L set to lower limit of detection divided by two (3.35 mg/L). Urine creatinine measured by enzymatic assay (Beckman Coulter) on an AU5400 analyzer within analytical range 88-44,200 micromol/L.
**Macroalbuminuria**	Urine albumin to creatinine ratio > 300mg/g	
**Microalbuminuria**	Urine albumin to creatinine ratio 30-300mg/g	
**End-stage renal disease**	http://biobank.ctsu.ox.ac.uk/showcase/field.cgi?id=42026	Centrally algorithmically defined by UK Biobank

**Heart failure**	ICD-9: 428, ICD-10: I50, I11.0, I13.0, I13.2	
**Type 2 diabetes**	Self-reported diagnosis of diabetes, current diabetes medication, physician diagnosis of type 2 diabetes, HbA1c > 6.5%	
**All-cause mortality**	http://biobank.ctsu.ox.ac.uk/showcase/field.cgi?id=40007	
**Cardiovascular mortality**	http://biobank.ctsu.ox.ac.uk/showcase/field.cgi?id=40001	Underlying primary cause of death ICD-10 I00-I99
**HbA1c**	http://biobank.ctsu.ox.ac.uk/showcase/field.cgi?id=30750	Measured by HPLC analysis on a Bio-Rad VARIANT II Turbo
**Body mass index**	http://biobank.ctsu.ox.ac.uk/showcase/field.cgi?id=21001	Calculated from height and weight measured during baseline assessment center visit.
**Current smoker**	http://biobank.ctsu.ox.ac.uk/showcase/field.cgi?id=20116	Answer "Current smoker"
**Ever smoker**	http://biobank.ctsu.ox.ac.uk/showcase/field.cgi?id=20116	Answers "Current smoker" + "Previous smoker"
**Serum cholesterol**	http://biobank.ctsu.ox.ac.uk/showcase/field.cgi?id=30690	Enzymatic assay (Beckman Coulter, UK); AU5800 Analyzer
**Serum HDL-cholesterol**	http://biobank.ctsu.ox.ac.uk/showcase/field.cgi?id=30760	Enzymatic Immuno-inhibition assay (Beckman Coulter); AU5800 analyzer
**Serum LDL-cholesterol**	http://biobank.ctsu.ox.ac.uk/showcase/field.cgi?id=30780	Enzymatic selective protection assay (Beckman Coulter); AU5800 Analyzer
**Serum triglycerides**	http://biobank.ctsu.ox.ac.uk/showcase/field.cgi?id=30870	Enzymatic assay (Beckman Coulter); AU5800 Analyzer

Table S2. Publicly available summary genome-wide association study results, Related to Figure 1 and Figure 2. Obtained as search results in PhenoScanner, or manual search of GWAS results for heart failure (HERMES Consortium) and kidney disease endpoints (CKDGen Consortium). We selected rs2107595 (chr7:19,009,765) as proxy if rs57301765 was not available (linkage disequilibrium in the European reference sample, R^2 0.965, D' 0.986, https://ldlink.nci.nih.gov/).

RSID	Hg38	A1	A2	Proxy	Trait	PMID/Source	Ethn	Beta	SE	P	N
**rs57301765**	chr7:19013110	A	G	rs2107595	Pulse pressure	26390057	Mixed	0.307	0.046	4.0E-11	320,251
**rs57301765**	chr7:19013110	A	G	rs2107595	Pulse pressure	28739976	EUR	0.291	0.063	4.0E-06	378,379
**rs57301765**	chr7:19013110	A	G	rs2107595	SBP	21909115	EUR	NR	NR	0.005	69,395
**rs57301765**	chr7:19013110	A	G	rs2107595	SBP	21909115	EUR	NR	NR	0.005	69,395
**rs57301765**	chr7:19013110	A	G	rs2107595	Aortic valve calcification	23388002	Mixed	NR	NR	0.728	6,942
**rs57301765**	chr7:19013110	A	G	rs2107595	DBP	21909115	EUR	NR	NR	0.767	69,395
**rs57301765**	chr7:19013110	A	G	-	Heart failure	HERMES*	EUR	0.026	0.011	0.015	956,557
**rs57301765**	chr7:19013110	A	G	-	eGFRcreat	CKDGen**	EUR	1e-04	5e-04	0.817	525,153
**rs57301765**	chr7:19013110	A	G	-	CKD	CKDGen**	EUR	0.013	0.013	0.322	402,682
**rs57301765**	chr7:19013110	A	G	-	eGFRcreat	CKDGen**	Mixed	-3e-04	4e-04	0.493	721,594
**rs57301765**	chr7:19013110	A	G	-	CKD	CKDGen**	Mixed	0.014	0.010	0.147	579,035
**rs57301765**	chr7:19013110	A	G	-	Microalbuminuria	CKDGen**	Mixed	-0.013	0.009	0.171	336,215
**rs57301765**	chr7:19013110	A	G	-	Urine ACR	CKDGen**	EUR	-0.005	0.003	0.047	533,701
**rs57301765**	chr7:19013110	A	G	-	Urine ACR	CKDGen**	Mixed	-0.006	0.003	0.037	549,054

*https://www.biorxiv.org/content/10.1101/682013v1; Data available here: http://www.broadcvdi.org/

**Summary data from different studies in CKDGen available here: https://ckdgen.imbi.uni-freiburg.de/

A1: effect allele, A2: other allele, Ethn: Ethnicity, AVC: aortic valve calcification, DBP: diastolic blood pressure, NR: not reported, PP: pulse pressure, SBP: systolic blood pressure, SE: standard error. Linkage disequilibrium between rs57301765 and rs2107595 in EUR reference r^2 0.9649, D-prime 0.9858

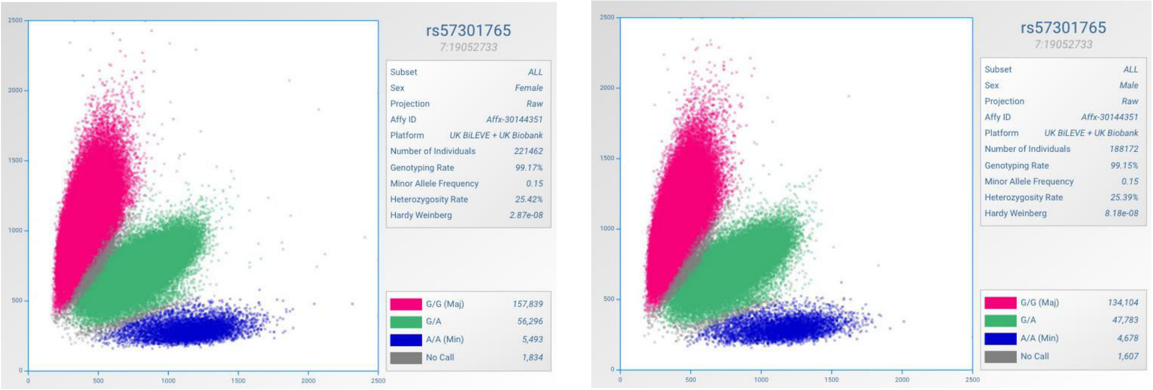

Figure S1. Cluster plots of genotype classes, Related to Figure 1 and Figure 2. Genotypes G/G, A/G, and A/A in 409,634 White British participants in the UK Biobank for women (left), and men (right) are shown as provided by *ScatterShot*.

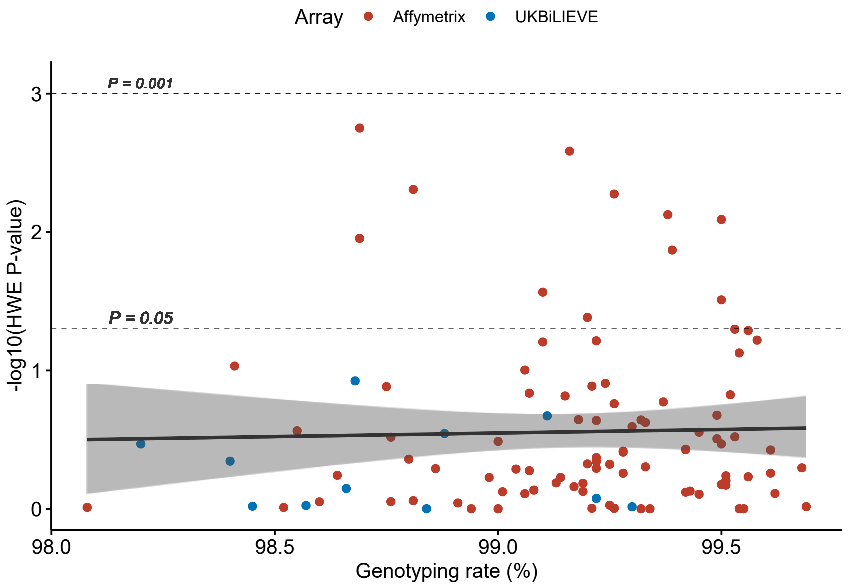

Figure S2. Scatterplot of -log$_{10}$(HWE P-value) against genotyping rate, Related to Figure 1 and Figure 2. Each dot represents one of the genotyping batches of the Affymetrix (red), or UKBiLIEVE arrays (blue). The line drawn through the dots represents the ordinary least squares best fit line with associated 95% confidence interval (shaded area).

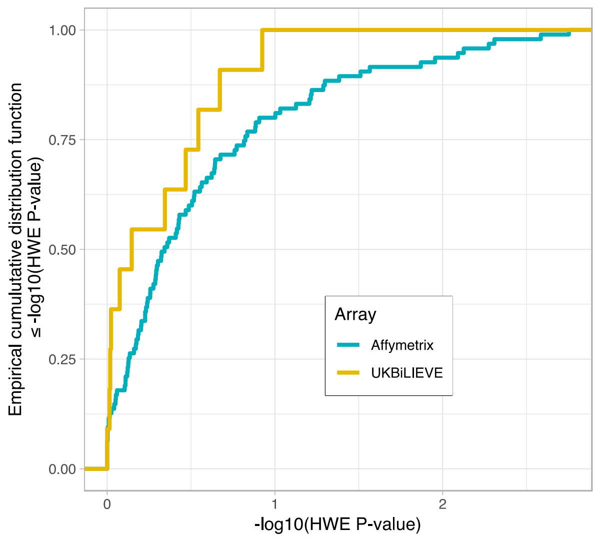

Figure S3. Cumulative -log$_{10}$(HWE P-value) distribution functions for batches of the Affymetrix and UKBiLIEVE array, Related to Figure 1 and Figure 2.

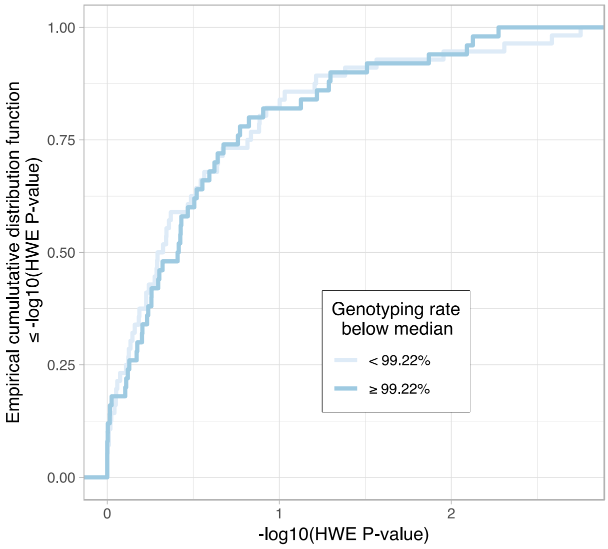

Figure S4. Cumulative -log10(HWE P-value) distribution functions for batches with genotyping rates above or below the median rate, Related to Figure 1 and Figure 2.
